# National Trends in the Safety Performance of Electronic Health Record Systems From 2009 to 2018

**DOI:** 10.1001/jamanetworkopen.2020.5547

**Published:** 2020-05-29

**Authors:** David C. Classen, A. Jay Holmgren, Zoe Co, Lisa P. Newmark, Diane Seger, Melissa Danforth, David W. Bates

**Affiliations:** 1 Division of Clinical Epidemiology, University of Utah School of Medicine, Salt Lake City; 2 Harvard Business School, Boston, Massachusetts; 3 Department of General Internal Medicine, Brigham and Women’s Hospital, Boston, Massachusetts; 4 Clinical and Quality Analysis, Partners Healthcare, Somerville, Massachusetts; 5 The Leapfrog Group, Washington, DC; 6 Harvard Medical School, Boston, Massachusetts

## Abstract

**Importance:**

Despite the broad adoption of electronic health record (EHR) systems across the continuum of care, safety problems persist.

**Objective:**

To measure the safety performance of operational EHRs in hospitals across the country during a 10-year period.

**Design, Setting, and Participants:**

This case series included all US adult hospitals nationwide that used the National Quality Forum Health IT Safety Measure EHR computerized physician order entry safety test administered by the Leapfrog Group between 2009 and 2018. Data were analyzed from July 1, 2018 to December 1, 2019.

**Exposure:**

The Health IT Safety Measure test, which uses simulated medication orders that have either injured or killed patients previously to evaluate how well hospital EHRs could identify medication errors with potential for patient harm.

**Main Outcomes and Measures:**

Descriptive statistics for performance on the assessment test over time were calculated at the overall test score level, type of decision support category level, and EHR vendor level.

**Results:**

Among 8657 hospital-years observed during the study, mean (SD) scores on the overall test increased from 53.9% (18.3%) in 2009 to 65.6% (15.4%) in 2018. Mean (SD) hospital score for the categories representing basic clinical decision support increased from 69.8% (20.8%) in 2009 to 85.6% (14.9%) in 2018. For the categories representing advanced clinical decision support, the mean (SD) score increased from 29.6% (22.4%) in 2009 to 46.1% (21.6%) in 2018. There was considerable variation in test performance by EHR.

**Conclusions and Relevance:**

These findings suggest that despite broad adoption and optimization of EHR systems in hospitals, wide variation in the safety performance of operational EHR systems remains across a large sample of hospitals and EHR vendors. Hospitals using some EHR vendors had significantly higher test scores. Overall, substantial safety risk persists in current hospital EHR systems.

## Key Points

**Question:**

How did safety performance of electronic health record systems (EHRs) change in the US from 2009 to 2018?

**Findings:**

In this case series using 8657 hospital-year observations from adult hospitals nationwide that used the National Quality Forum Health IT Safety Measure, a computerized physician order entry and EHR safety test, from 2009 to 2018, mean scores on the overall test increased from 53.9% in 2009 to 65.6% in 2018. There was considerable variation in test performance by hospital and EHR vendor.

**Meaning:**

These findings suggest that, despite broad adoption and optimization of EHR systems in hospitals, wide variation in the safety performance of operational EHR systems remains across a large sample of hospitals and EHR vendors, and serious safety vulnerabilities persist in these operational EHRs.

## Teaser

This case series examines the safety performance of computerized provider order entry in electronic health record systems in US hospitals from 2009 to 2018.

## Introduction

The Institute of Medicine’s 1999 report, “To Err is Human” brought into the public eye the issue of medical errors in modern medicine and estimated that approximately 98 000 deaths and 1 000 000 inpatient injuries occur annually in the US because of medical errors.^1^ That report noted that medication safety problems were the most frequent cause of preventable harm and also recommended broad adoption of electronic health records (EHRs) with computerized physician order entry (CPOE) and clinical decision support (CDS) to improve medication safety. More recent reports suggest that medication safety and overall safety problems are still unacceptably high, despite the broad adoption of these EHR systems.^1^

In the decades that followed, implementation of EHRs with the use of CPOE and associated CDS accelerated. This effort was driven in part by the Health Information Technology for Economic and Clinical Health Act, which drove the development of meaningful use incentives for EHR adoption.^2^ A subsequent Institute of Medicine report on patient safety and health information technology (IT) published in 2012 reviewed the progress in adoption of EHRs and found the adoption of EHRs had not yet led to expected significant improvements in medication safety.^3^ That report made recommendations about improving medication safety through more effective development and use of EHRs with CDS. A specific recommendation was that health care organizations should adopt a best practice commonly used in other industries, ongoing testing of safety performance of software in actual use, which has rarely been done in health care.^3^ The particular concern was that organizations purchase EHRs and medication safety tools from separate vendors and have great latitude in how they implement and maintain them, so substantial variation in safety performance could be present.

The Institute of Medicine’s report^3^ specifically highlighted an initiative developed to improve medication safety through health information technology, the Leapfrog CPOE EHR evaluation tool. Leading patient safety experts working with the Leapfrog Group developed an independent, inexpensive, and standardized tool embedded in Leapfrog’s annual voluntary hospital survey to evaluate the performance of EHR systems in reducing adverse drug events (ADEs) using simulated real patients and real-world inpatient medication orders.^4,5,6,7,8,9^ The tool has been used mainly in general acute care hospitals; more than 1800 hospitals used it in 2018. In a 2013 study of many hospitals,^7^ scores using this EHR evaluation tool were strongly correlated with rates of preventable ADEs in included hospitals, with 4 fewer preventable ADEs per 100 admissions for every 5% increase in overall score.

We used data collected through this Leapfrog CPOE EHR evaluation tool from a large national sample of hospitals during a 10-year period to address 3 research questions. First, we evaluated progress over 10 years of overall safety performance of EHRs to prevent potential ADEs. Second, we assessed hospital EHR safety performance for specific subcategories of potential ADEs. Third, we examined the associations of EHR vendor with safety performance on the Leapfrog CPOE EHR evaluation tool.

## Methods

This study was reviewed and approved by the University of Utah institutional review board. The need for informed consent was waived because this study did not involve any real patients or real patient data.

### Design of the CPOE EHR Evaluation Tool

The CPOE EHR evaluation tool was designed by investigators at the University of Utah and the Brigham and Women’s Hospital and has been used by the Leapfrog Group, whose mission is to encourage “giant leaps” in patient safety and quality.^10,11^ The CPOE EHR evaluation tool is included as part of the annual Leapfrog Hospital Survey, which is a free, annual survey distributed nationally to US hospitals with results reported publicly.^10^ The Leapfrog CPOE EHR evaluation tool is endorsed by the National Quality Forum as part of their “Safe Practices for Better Healthcare” report, which includes a CPOE standard.^12^ This test focuses on medication safety, still the leading cause of harm due to medical errors in hospital patients, but it does not represent all the ways EHRs can improve safety

The Leapfrog CPOE EHR evaluation tool is a simulation that uses real-world test patients and medication orders to mimic the experience of a physician writing orders for actual patients to evaluate EHR safety performance. The test patients and orders were developed by a group of experts on ADEs and CPOE CDS to test how effectively hospital CPOE and EHR systems alert clinicians to potential ADEs. The test orders were developed specifically to assess whether orders that were likely to cause the most serious patient harm would be identified; almost all of these scenarios were drawn from real-world incidents of preventable ADEs from real patients who experienced injuries or death.^4,5,6^ The order types are divided into 2 categories, orders with potential adverse events prevented by basic CDS (ie, drug-allergy, drug-route, drug-drug, drug-dose for single doses, and therapeutic duplication contraindications) and those that would require advanced CDS (ie, drug-laboratory, drug-dose for daily doses, drug-age, drug-diagnosis, and corollary orders contraindications).^5^ The primary outcome measure was whether the hospital CPOE EHR system correctly generated an alert, warning, or soft or hard stop after entering a test order that could have caused an ADE.^5^ The test had its content updated in 2010 and 2017, primarily to adjust to changes in drug formularies commonly used in hospitals; the performance of the test and the scoring of the test were not changed in either of these updates.^9^

To participate in this high fidelity test, a hospital representative downloads and enters a set of test patients with detailed profiles, including diagnoses, laboratory test results, and other information, into their EHR as real patients would be admitted to their hospital. A clinician with experience using the institution’s CPOE EHR application then enters test medication orders into the EHR for these test patients who have been admitted to the hospital and records in detail how the EHR responds, including what, if any, CDS in the form of alerts, messages, guidance, soft or hard stops, or other information are presented and whether the order is blocked or allowed to be entered in the EHR system. A hospital representative then enters all these responses in detail into the CPOE EHR evaluation tool, an overall score is immediately calculated, and a report is generated for the hospital. In addition to the overall score, 10 categorical scores are then presented to the hospital, with categories such as allergy, drug interaction, renal dosing, excess daily dosing, wrong drug route, disease-drug, drug serum level–checking, and drug-age contraindications. To ensure the test is taken as intended and to prevent gaming of the system, a number of control orders are included that are not expected to invoke any alerts. The entire process is timed so that no hospital can take longer than 6 hours total to complete the test, but most hospitals complete it in 2 to 3 hours.^4,5,6,7,8,9^ Hospitals that exceed this timed threshold or report too many alerts on control or normal orders are disqualified, although this amounts to less than 1% of hospitals each year.

### Data Collection and Sample

Our sample included hospitals who took the Leapfrog Hospital Survey, including the CPOE EHR evaluation tool, in at least 1 year from 2009 through 2018. Hospitals that began taking the test but did not fully complete it in a given year were marked as incomplete and excluded for that year. If a hospital took the test a maximum of 2 times in a single year, we kept the highest overall scoring test. If a test order was not on the hospital’s formulary or otherwise not prescriptible, it was omitted from the numerator and denominator used to calculate scores. However, if a hospital’s EHR system did not have a specific alert functionality for that category, the test orders were not omitted.

Our final analytic sample included all hospitals with at least 1 completed test from 2009 through 2018 regardless of how many years they completed the test. We then linked these results with data from the American Hospital Association Annual Survey from 2009 to 2018 to capture hospital demographic information.^13^ Hospitals were matched based on their Medicare identification number in each year.

### EHR Vendor

To determine a hospital’s EHR vendor, we used self-reported data from the hospital when they used the CPOE EHR evaluation tool. Each vendor with more than 100 observations in our data was kept as a separate vendor, while all vendors with fewer than 100 observations each were categorized as other. Vendor names were anonymized per our data use agreement.

### Hospital Characteristics

We selected a set of hospital characteristics that we expected to be associated with hospital CPOE EHR performance based on previous studies of health IT adoption.^9,10,11,14,15,16,17,18,19,20^ These included size (measured by number of beds), membership in a health care system, teaching status, ownership (including private for-profit, private nonprofit, and public nonfederal hospitals), and urban or rural location, as well as geographic region within the United States based on US census areas (ie, Northeast, West, Midwest, and South).

### Statistical Analysis

We first calculated a set of sample descriptive statistics, including the mean and SD, for hospital demographic characteristics of our sample, including EHR vendor. Next, we calculated mean CPOE EHR assessment test scores from 2009 through 2018 by the basic and advanced CDS categories. Then, we calculated CPOE EHR performance scores over time from 2009 to 2018 by each specific order category. We then created a bivariate comparison of CPOE EHR performance scores by EHR vendor.^5,6,7,8,9^

We also developed a multivariate ordinary least squares regression model with hospital CPOE EHR test scores as our dependent variable and hospital EHR vendors as the independent variable. Our model also included hospital demographic characteristics as controls, year–fixed effects to account for secular improvement trends over time, and robust SEs clustered at the hospital level.^9^

Analyses were performed using Stata statistical software version 16 (StataCorp). *P* values were 2-sided, and statistical significance was set at .05. Data were analyzed from July 1, 2018 to December 1, 2019.

## Results

### Sample Characteristics

Our analytic sample included data from 2314 hospitals with at least 1 year of test results, for a total of 8657 unique hospital-year observations. Hospitals included were a large sample of US hospitals (Table 1). Most observations were from medium-sized hospitals, with 100 to 399 beds (4429 observations [51.2%]), followed by large-sized hospitals with more than 400 beds (2727 observations [31.5%]) and small hospitals with fewer than 100 beds (1501 observations [17.3%]). Most observations were from hospitals that were part of a health care system (6117 observations [70.7%]), and 3813 observations (44.0%) were from teaching hospitals. Most hospital-year observations were in an urban area (6044 observations [69.8%]). Private nonprofit ownership was the most common ownership model (5326 observations [61.5%]), followed by private for-profit (1494 observations [17.3%]) and public nonfederal (780 observations [9.0%]). Regarding region, 2698 observations (31.2%) were from hospitals in the Southern US, while 1870 observations (21.6%) were from hospitals in the West, 1548 observations (17.9%) were from hospitals in the Northeast, and 1484 observations (17.1%) were from hospitals in the Midwest. The total number of hospitals taking the test increased from 157 hospitals in 2009 to 1812 hospitals in 2018. The breakdown of how many years hospitals took the test is presented in the eFigure in the Supplement.

**Table 1.** Hospital Characteristics

Characteristic	Hospital-year observations, No. (%)
EHR vendor	
A	2620 (30.7)
B	2199 (25.7)
C	1996 (23.4)
D	514 (6.0)
E	352 (4.1)
F	225 (2.6)
G	141 (1.7)
H	111 (1.3)
Other	386 (4.6)
Hospital size (beds)	
Small (<100)	1501 (17.3)
Medium (100-399)	4429 (51.2)
Large (≥400)	2727 (31.5)
Organizational characteristics	
Member of a health care system	6117 (70.7)
Teaching hospital	3813 (44.0)
Location	
Rural	2613 (30.2)
Urban	6044 (69.8)
Ownership	
Private nonprofit	5326 (61.5)
Private for-profit	1494 (17.3)
Public nonfederal	780 (9.0)
Geographic region	
Northeast	1548 (17.9)
West	1870 (21.6)
Midwest	1484 (17.1)
South	2698 (31.2)

Abbreviation: EHR, electronic health record.

### CPOE EHR Assessment Scores Over Time

**Figure 1.**
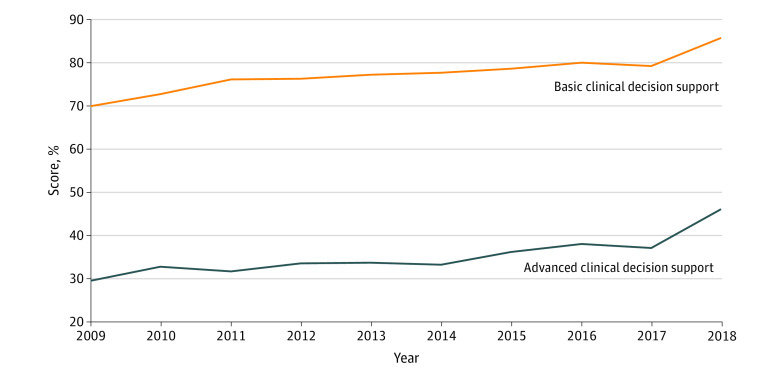
Basic and Advanced Clinical Decision Support Test Scores Over 10 Years

The overall mean (SD) total score increased from 53.9% (18.3%) in 2009 to 65.6% (15.4%) in 2018. Mean (SD) hospital score for the categories representing basic CDS increased from 69.8% (20.8%) in 2009 to 85.6% (14.9%) in 2018 (Figure 1). For the categories representing advanced CDS, the mean (SD) score increased from 29.6% (22.4%) in 2009 to 46.1% (21.6%) in 2018.

**Figure 2.**
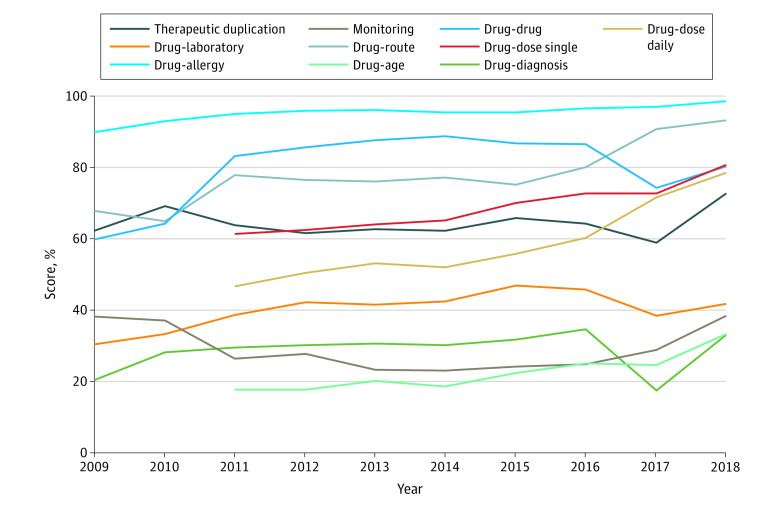
Category Test Scores Over Time

Examining individual categories’ score during the 10-year period evaluated, the highest performing category was drug-allergy in each year, increasing from 92.9% (14.6%) in 2009 to 98.4% (7.2%) in 2018 (Figure 2). The lowest performing category throughout the study was drug-diagnosis contraindications, with a mean (SD) score of 20.4% (27.4%) in 2009 and 33.2% (35.8%) in 2018. The category with the greatest improvement was drug-age contraindications, from a mean (SD) score of 17.7% (29.6%) in 2011 when it was added to the test to 33.2% (38.4%) in 2018, and the least improved category was drug-allergy contraindications.

### EHR Vendor Performance

**Figure 3.**
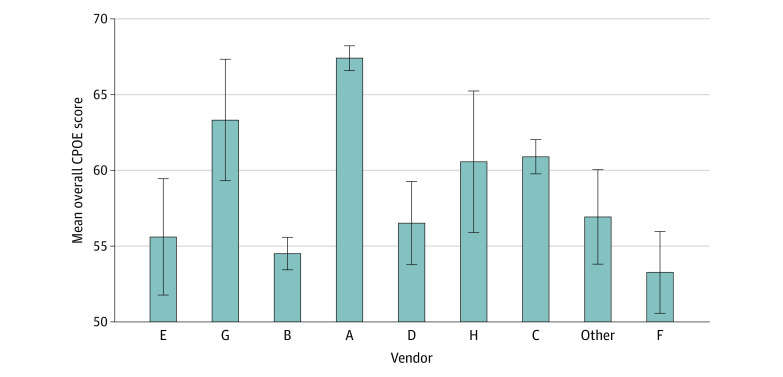
Summary of Hospital Overall Scores by Electronic Health Record Vendor CPOE indicates computerized physician order entry; Whiskers, SD.

Our sample included hospitals using 30 different EHRs, 8 of which had more than 100 observations and were kept distinctly identified as vendors A through H, while the rest were all classified as other vendor. We summarize the leading vendors by number of hospitals using them and mean test score in Figure 3. The largest vendor, vendor A, had the highest overall score (67.4%) for the overall test period, followed by vendor G (63.2%), vendor C (60.8%), vendor H (60.6%), other vendors (56.8%), vendor D (56.6%), vendor E (55.6%), vendor B (54.5%), and finally, vendor F (53.4%). More detailed EHR descriptive statistics are in the eTable in the Supplement.

In the multivariate regression analysis, we found that when controlling for observable hospital characteristics, including size, teaching status, ownership model, system membership, and location, 3 vendors had higher CPOE assessment test scores: vendor A (β = 11.26; 95% CI, 8.10-14.42; *P* < .001), vendor G (β = 5.49; 95% CI, 0.77-7.81; *P* = .02), and vendor C (β = 3.57; 95% CI, 0.32-6.81; *P* = .03) (Table 2). In determining how much variation in medication safety performance was explained by vendor, we calculated the partial *R*^2^ of regressing the vendors against the overall Leapfrog score and found that vendor choice explained 9.9% of variation in performance, while vendor choice combined with observable hospital characteristics from our full regression explained 14.6% of the variation (Table 2).

**Table 2.** Hospital and EHR Vendor Correlations

Variable	β (95% CI)	*P* value
EHR vendor		
Other	[Reference]	NA
A	11.26 (8.10 to 14.42)	<.001
B	−2.21 (−0.54 to 0.99)	.18
C	3.57 (0.32 to 6.81)	.03
D	0.47 (−3.59 to 4.52)	.82
E	−1.41 (−5.97 to 3.15)	.55
F	−3.38 (−7.45 to 0.68))	.10
G	5.49 (0.77 to 10.20	.02
H	2.41 (−2.98 to 7.81)	.38
Vendor only partial *R*^2^	0.099	NA
*R*^2^ including hospital characteristics controls	0.146	NA

Abbreviations: EHR, electronic health record; NA, not applicable.

## Discussion

This case series used national, longitudinal data to track the safety performance of hospital EHRs during a 10-year period using an objective EHR safety test that is a National Quality Forum–endorsed measure of health IT safety. To our knowledge, this is the first large-scale study of operational hospital EHR medication safety over time and the first to examine both within- and across-vendor variation. We found that overall safety performance increased modestly, while the number of institutions taking the test has increased 10-fold. Improvements in basic CDS were far greater than in advanced CDS, consistent with other studies.^5,8,9^ Basic CDS capabilities have achieved high performance across multiple domains, but performance has been mixed in other areas, such as therapeutic duplication contraindications. This may be partially attributable to hospitals relying on dispensing pharmacists to prevent these errors. However, the scenarios used in the test represent real-world cases in which patients were injured or killed even with pharmacist review. Relying on dispensing pharmacists alone removes an important layer of safety checks.^3^

The potential ADE categories labeled as advanced CDS capabilities did improve modestly, but enormous potential remains. Only 1 category, drug dose daily contraindications, was at a level consistent with the basic CDS categories. Some evidence suggests that advanced CDS, such as renal dosing contraindications, may be the most important type for improving medication safety.^21^ While most advanced CDS categories have improved little during the last 10 years, high performance is possible. Several hospitals have achieved a perfect score in all the categories of the test, representing each of the 9 leading EHR vendors being used by at least 1 hospital with a perfect score. Given that many of the ADEs potentially prevented by advanced CDS are significant areas of focus for many hospitals, such as managing polypharmacy for older patients, this slow progress is particularly concerning. Policy makers may wish to incentivize hospital safety improvements by including measures of CPOE EHR safety performance as quality benchmarks in future health care payment reform programs.

These results also reveal important associations in safety performance both within and across EHR vendors. It is often assumed by hospitals that the bigger the EHR vendor in terms of market share, the less the likelihood of safety performance problems. However, recent examples from other industries, notably the failure of the Boeing 737 airplane caused by software malfunction, underscores the risks of this assumption.^22^ In our results, the most popular vendor, vendor A, did have the highest mean safety scores, but there was variability among vendor A’s implementations, and the second-most popular vendor had among the lowest safety scores, with many smaller EHR vendors in the top 5 in overall safety performance. Additionally, while we found significant variation in safety performance across vendors, there was also heterogeneity within vendors, suggesting that both technology and organizational safety culture and processes are important contributors to high performance.^18,23^

Hospitals, EHR vendors, and policy makers can seek to improve safety performance in several ways. First, hospitals should consider performing some type of CPOE safety evaluation at least annually or after upgrades and work to address identified shortcomings. Continuous assessments are also critical to identify unanticipated problems that may occur as systems are updated and customized. They should also share these results with their EHR vendor to help these vendors create safer products, as safety is a shared responsibility between vendors and hospitals.^3^ Policy makers may wish to include CPOE safety evaluation scores in their suite of process quality measures reported publicly.

### Limitations

Our study has several limitations. First, while we have a large sample of hospitals from across the US, we were limited to those that completed the Leapfrog Annual Practices Survey. This may result in a sample that is not representative of all US hospitals; if selection bias exists, it is likely that hospitals that select to use this evaluation are more interested in safety and improvement, which would suggest that the true safety performance of US hospitals is worse than our results show. Second, while efforts were made to keep the CPOE EHR assessment tool consistent throughout the study, refreshes of the test content occurred in 2011 and 2017; however, there were no changes in scoring at the overall or category level. Third, our study measured process quality as whether the CPOE system performed correctly, rather than assessing direct patient outcomes, although better performance on the test has been shown to be correlated with lower rates of actual preventable ADEs in a study of hospitals.^7^ Fourth, the Leapfrog CPOE EHR evaluation tool is a simulation of patients and medication orders, the patient scenarios and orders are based on real patients and real ADEs and use the institution’s active EHR; however, there may be other factors that would affect performance in real patients.

## Conclusions

The findings of this case series suggest that although hospitals across the US have nearly universally adopted EHRs during the past 2 decades, the associations of these systems with safety are still mixed. These EHR systems are large, complex, and constantly evolving, yet they are largely unregulated with respect to safety. Using a standardized, federally endorsed health IT safety measure, we have found that these systems meet the most basic safety standards less than 70% of the time and that these systems have only modestly increased their safety performance during a 10-year period, leaving critical deficiencies in these systems to detect and prevent critical safety issues. We also found associations between EHR vendor and medication safety performance. These findings emphasize the need to continually evaluate the safety performance of these systems in actual use with ongoing operational testing.

## Notes

Supplement. **eFigure.** Histogram of Number of Years Taken by Hospitals **eTable.** Descriptive Statistics by EHR Vendor
