# Supplementary material for: National Trends in the Safety Performance of Electronic Health Record Systems From 2009 to 2018
Source: JAMA Netw Open. 2020 May 29;3(5):e205547. doi: 10.1001/jamanetworkopen.2020.5547 (PMC7260621; doi:10.1001/jamanetworkopen.2020.5547)
Supplement: Supplement. — eFigure. Histogram of Number of Years Taken by Hospitals eTable. Descriptive Statistics by EHR Vendor [file jamanetwopen-3-e205547-s001.pdf]

Supplementary Online Content

Classen DC, Holmgren AJ, Co Z, et al. National trends in the safety performance of electronic health record systems from 2009 to 2018. *JAMA Netw Open*. 2020;3(5):e205547. doi:10.1001/jamanetworkopen.2020.5547

eFigure. Histogram of Number of Years Taken by Hospitals
eTable. Descriptive Statistics by EHR Vendor

This supplementary material has been provided by the authors to give readers additional information about their work.

© 2020 Classen DC et al. *JAMA Network Open.*

eFigure. Histogram of Number of Years Taken by Hospitals

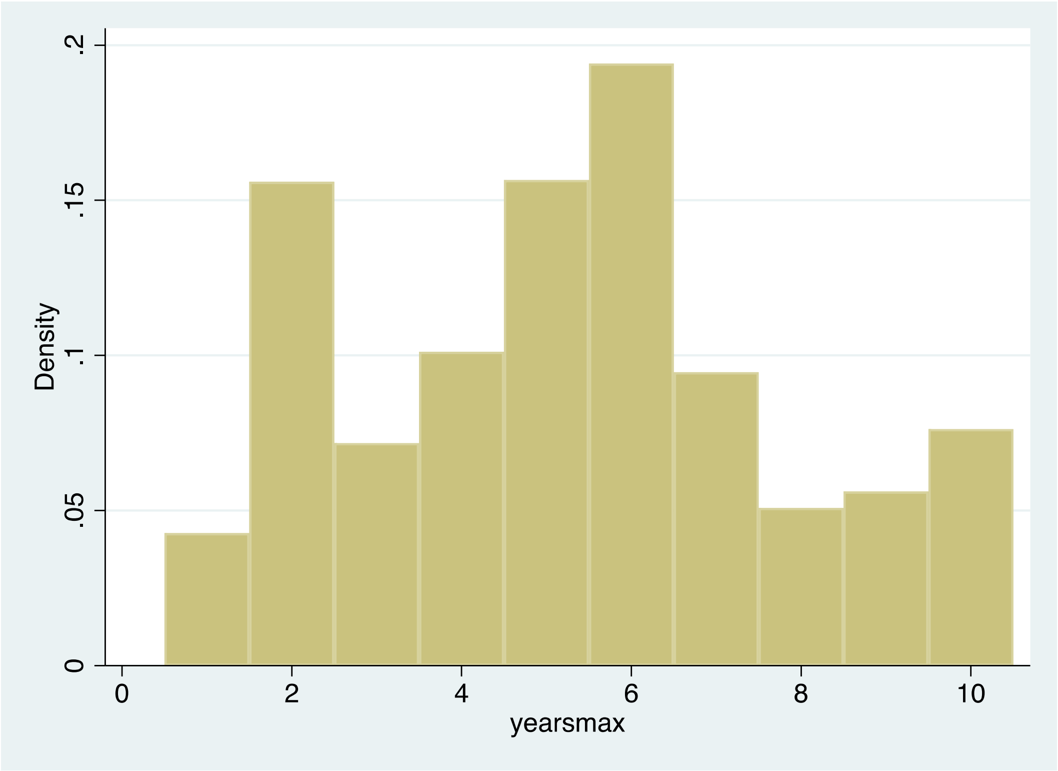

© 2020 Classen DC et al. *JAMA Network Open.*

eTable. Descriptive Statistics by EHR Vendor

EHR Vendor	Mean	N	Minimum	Maximum	Standard Deviation
Vendor E	55.60	352	12.77	100.00	20.37
Vendor G	63.32	141	17.73	97.44	18.07
Vendor B	54.51	2199	0.00	100.00	17.14
Vendor A	67.40	2620	0.00	100.00	12.83
Vendor D	56.52	514	0.00	100.00	18.39
Vendor H	60.57	111	0.00	100.00	18.98
Vendor C	60.90	1996	17.14	100.00	16.07
Other Vendor	56.93	386	12.77	98.58	18.39
Vendor F	53.27	225	13.83	100.00	15.25

© 2020 Classen DC et al. *JAMA Network Open.*
